# Glomerular Filtration Rate by Differing Measures in Predicting Atrial Fibrillation Recurrence After Ablation

**DOI:** 10.31083/RCM42848

**Published:** 2025-12-18

**Authors:** Fangyuan Luo, Zhe Wang, Jiajie Yin, Danni Wu, Song Wu, Jianzeng Dong, Yingwei Chen, Xianlun Li

**Affiliations:** ^1^China-Japan Friendship Hospital (Institute of Clinical Medical Sciences), Chinese Academy of Medical Sciences & Peking Union Medical College, 100029 Beijing, China; ^2^Department of Integrative Medicine Cardiology, China-Japan Friendship Hospital, 100029 Beijing, China; ^3^Department of Cardiology, Beijing Anzhen Hospital, Capital Medical University, 100029 Beijing, China; ^4^Department of Cardiology, The First Affiliated Hospital of Zhengzhou University, 450052 Zhengzhou, Henan, China

**Keywords:** glomerular filtration rate, creatinine, cystatin C, atrial fibrillation, radiofrequency ablation, recurrence

## Abstract

**Background:**

Significant differences often exist between estimated glomerular filtration rates (eGFR) calculated using various biomarkers. However, the relationship between these eGFR methods and atrial fibrillation (AF) recurrence after radiofrequency catheter ablation (RFCA) remains unclear.

**Methods:**

Thus, this study employed a retrospective analysis of 523 patients with AF who underwent an initial RFCA between July 2019 and October 2022. The eGFR was calculated using three methods based on the Chronic Kidney Disease Epidemiology Collaboration (CKD-EPI) formula: serum creatinine (eGFRcr), serum cystatin C (eGFRcys), and a combination of both (eGFRcrcys). Cox regression models were used to explore the relationship between eGFR and AF recurrence.

**Results:**

Over a 1-year follow-up period, 174 (33.3%) patients experienced AF recurrence after RFCA. Multivariable Cox regression analysis indicated that only eGFRcys showed a consistent, significant inverse association with AF recurrence (hazard ratio (HR) = 0.990, 95% confidence interval (CI): 0.982–0.998, *p *= 0.019). In contrast, eGFRcrcys showed borderline significance after full adjustment (*p *= 0.067). Meanwhile, stratifying by optimal cutoff values identified an association for eGFRcys ≤64.280 mL/min/1.73 m^2^, and eGFRcrcys ≤76.093 mL/min/1.73 m^2^ with significantly higher recurrence risks after full adjustment (*p *= 0.008 and *p* = 0.036, respectively). Additionally, incorporating eGFRcys or eGFRcrcys into the baseline risk model led to a greater improvement in predictive accuracy than adding eGFRcr.

**Conclusions:**

The association between eGFR and AF recurrence after ablation appears to vary depending on the measurement methods; eGFRcys seems to provide the most reliable information. Incorporating eGFRcys into the pre-ablation risk stratification may enhance patient management and improve outcomes for patients undergoing AF ablation.

## 1. Introduction

Atrial fibrillation (AF) is a major contributor to stroke, heart failure, various other complications, and mortality [1, 2]. Radiofrequency catheter ablation (RFCA) has become an important treatment approach; however, recurrence rates after ablation remain a significant clinical challenge [3]. Prompt and refined risk stratification is crucial for enhancing outcomes in patients with AF after ablation. Chronic kidney disease (CKD) is highly prevalent and has been increasingly recognized as an additional, independent risk factor for cardiovascular disease (CVD) [4, 5]. Of particular concern, AF occurs in up to 18% of patients with CKD, underscoring the complex interplay between renal dysfunction and cardiac arrhythmogenesis [6].

As a key measure of kidney function, the estimated glomerular filtration rate (eGFR) is commonly calculated using the Chronic Kidney Disease Epidemiology Collaboration (CKD-EPI) formula, a widely used approach that has traditionally relied on serum creatinine (eGFRcr) as its main parameter [7, 8]. However, mounting evidence suggests that incorporating cystatin C, either alone (eGFRcys) or in combination with creatinine (eGFRcrcys), enhances the accuracy of kidney function assessment and is more strongly associated with the risk of end-stage kidney disease, CVD, and mortality [9, 10, 11]. Interestingly, prior studies indicate that lower eGFRcys is strongly linked to incident AF, whereas eGFRcr often shows a weaker or inconsistent relationship [12, 13]. However, whether these different eGFR measures have distinct impacts on AF recurrence following RFCA remains unknown.

In the present study, we compared three eGFR estimation methods, including eGFRcr, eGFRcys, and eGFRcrcys, for their ability to predict AF recurrence after the first RFCA procedure. Given the burden of AF and CKD, clarifying which eGFR measures offer greater prognostic accuracy could enhance pre-ablation risk stratification strategies and guide more individualized patient management.

## 2. Methods

### 2.1 Study Participants

**Fig. 1.**
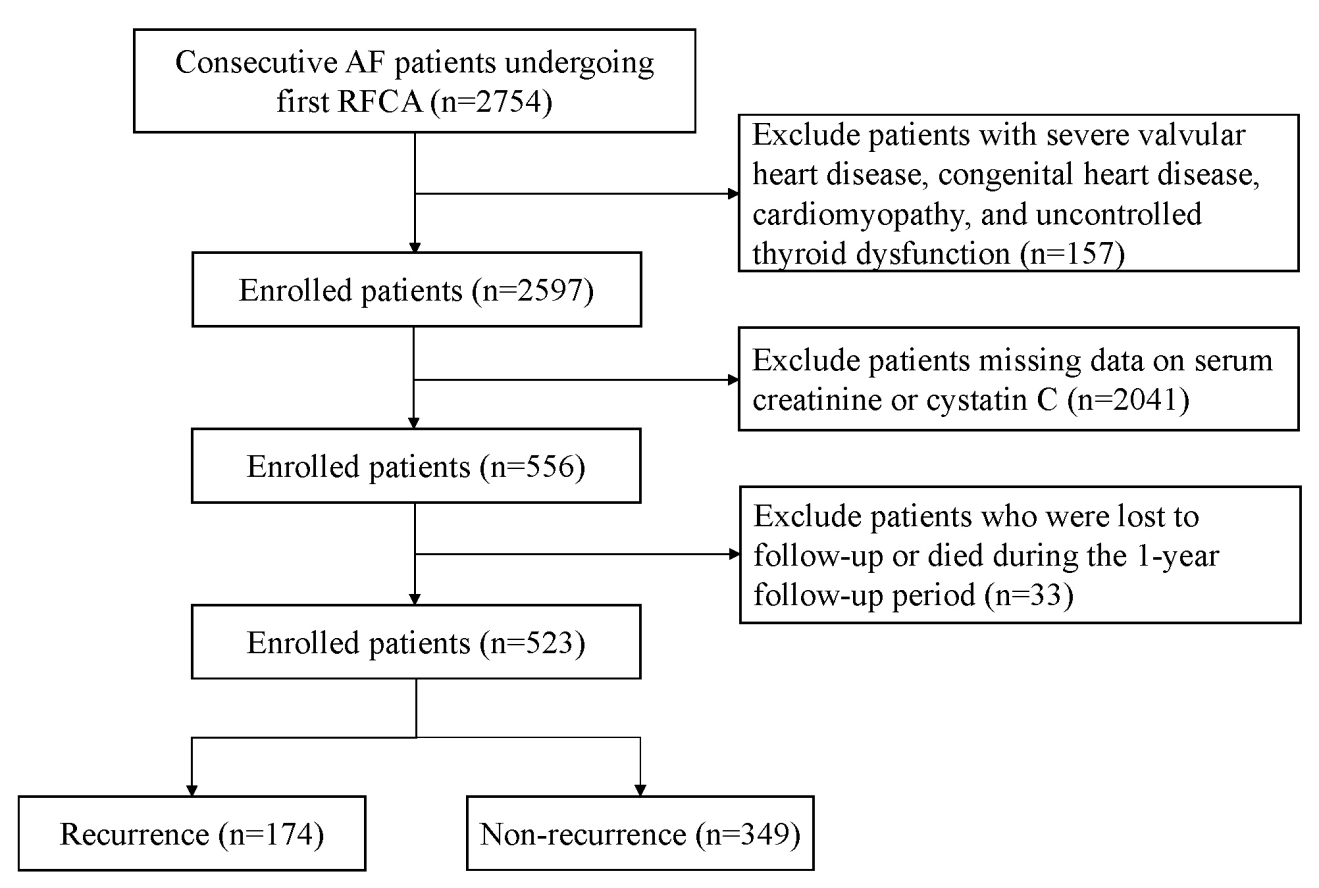
**The flowchart illustrates the selection of patients**. AF, atrial fibrillation; RFCA, radiofrequency catheter ablation.

We performed a retrospective analysis of patients with AF who underwent their initial RFCA at the First Affiliated Hospital of Zhengzhou University and the China-Japan Friendship Hospital between July 2019 and October 2022. The inclusion criteria were hospitalization for initial RFCA and available pre-ablation measurements of serum creatinine and cystatin C. We excluded patients with severe valvular heart disease, uncontrolled thyroid dysfunction, congenital heart disease, or cardiomyopathy, as well as those who were lost to follow-up or died during the follow-up period. This research followed the principles outlined in the Declaration of Helsinki and obtained approval from the ethics committees of the two hospitals (Approval Nos. 2023-KY-0327 and 2022-KY-043). The study design and participant flow are illustrated in Fig. 1.

### 2.2 Data Collection and Definitions

The data included various modules, such as clinical characteristics, comorbidities, medications, laboratory tests, and echocardiographic assessments. The duration of AF was determined by calculating the time from the initial diagnosis to the date of RFCA. Persistent AF was defined as AF that lasts longer than seven days. Complications, and preoperative medications, including amiodarone, angiotensin converting enzyme inhibitors (ACEI)/angiotensin receptor blocker (ARB), and statins, were extracted from the electronic medical records. All laboratory measurements were derived from fasting blood samples drawn from the patient’s peripheral veins prior to the ablation procedure. Echocardiographic measurements, including ejection fraction (EF), left ventricular end-diastolic dimension (LVED), and left atrial diameter (LAD), were recorded before the ablation. The ablation methods employed for each patient were documented in the ablation records.

### 2.3 Assessment of Kidney Function

The CKD-EPI equations, recommended by Kidney Disease: Improving Global Outcomes (KDIGO), are widely utilized for estimating eGFR [14]. In this study, we calculated eGFR using three methods based on the CKD-EPI formula: serum creatinine (eGFRcr), serum cystatin C (eGFRcys), and a combination of both (eGFRcrcys) [8, 9]. To classify kidney function, we used a threshold of 60 mL/min/1.73 m^2^, as an eGFR below this value is a widely recognized indicator of reduced kidney function in population-based research. The specific calculation formula is as follows:

eGFRcr = 142 × min(Scr/k,1)^α^ × max(Scr/k,1)^-1.200^ × 0.9938^age^ × 1.012 (if female) where Scr is serum creatinine, k is 0.7 for females and 0.9 for males, and α is –0.241 for females and –0.302 for males.

eGFRcys = 133 × min(Scys/0.8,1)^-0.499^ × max(Scys/0.8,1)^-1.328^ × 0.9962^age^ × 0.932 (if female) where Scys is serum cystatin C.

eGFRcrcys = 135 × min(Scr/k,1)^α^ × max(Scr/k,1)^-0.544^ × min(Scys/0.8,1)^-0.323^ × max(Scys/0.8,1)^-0.778^ × 0.9961^age^ × 0.963 (if female) where Scr is serum creatinine, Scys is serum cystatin C, k is 0.7 for females and 0.9 for males, and α is –0.219 for females and –0.144 for males.

### 2.4 Procedure and Management of Catheter Ablation

The RFCA technique has been previously detailed [15]. Briefly, all patients underwent RFCA guided by an electroanatomical mapping system (CARTO, Biosense Webster, Diamond Bar, CA, USA). After a three-dimensional reconstruction of the left atrium, circumferential pulmonary vein isolation was performed in all patients with AF. In persistent AF, additional linear ablations (roof line, mitral isthmus, and cavotricuspid isthmus) were routinely performed, and complex fractionated atrial electrogram ablation was considered if necessary. Superior vena cava (SVC) isolation was applied if induced tachycardia suggested an origin in the SVC or if active SVC potentials were detected. Bidirectional conduction block of all lines was confirmed immediately and reassessed after a 30-minute observation. If the pulmonary veins showed reconnection, re-isolation was done. When AF persisted despite these measures, sinus rhythm was restored using synchronized direct current cardioversion.

After the procedure, unless contraindicated, all patients received oral anticoagulant therapy for up to three months, along with a short-term regimen of antiarrhythmic drugs to reduce early recurrence.

### 2.5 Follow‑up and Outcomes

The primary outcome of this study was the recurrence of AF within one year following ablation. Recurrence was identified as any AF, atrial flutter, or atrial tachycardia episode exceeding 30 seconds, documented via electrocardiogram or 24-hour Holter monitoring following a 3-month blanking period [16, 17]. Patients were scheduled for follow-up assessments at 3, 6, 9, and 12 months, conducted either through outpatient visits or telephone consultations. At each interval, electrocardiogram and 24-hour Holter monitoring were recommended. Additionally, patients experiencing symptoms indicative of AF recurrence were advised to undergo immediate electrocardiogram and 24-hour Holter monitoring for confirmation.

### 2.6 Statistical Analysis

Continuous variables are expressed as the mean ± standard deviation or median (interquartile range), depending on distribution normality, and analyzed using the *t*-test or Mann-Whitney test. Categorical variables are summarized as frequencies and percentages, with comparisons made using the χ^2^ test where applicable. Event-free survival post-ablation was evaluated using Kaplan-Meier curves and the log-rank test.

Univariate and multivariate Cox regression analyses were conducted to investigate the relationship between different eGFR calculation methods and AF recurrence. Variables that showed statistical significance in the univariate analysis (*p* < 0.05) as well as those deemed clinically relevant were incorporated into the multivariate analysis model. Three models were used to address potential confounders: Model 1 did not account for any adjustments; Model 2 adjusted for age, sex, and body mass index (BMI); and Model 3 additionally incorporated variables identified in univariate Cox regression with *p* < 0.05 (AF type, AF duration ≥2 years, hemoglobin, LAD, and CHADS_2_ score). A collinearity test was conducted for each model to avoid overfitting. Hazard ratios (HRs) and 95% confidence intervals (CIs) were reported.

Receiver operating characteristic (ROC) curves, including the area under the curve (AUC), were employed to assess the predictive value of different eGFR measurement methods, both alone and in combination with a baseline risk model (age, sex, BMI, AF type, AF duration ≥2 years, hemoglobin, LAD, and CHADS_2_ score). The incremental predictive value of introducing eGFR calculation methods into the baseline model was assessed with the C-statistic, integrated discrimination improvement (IDI), and continuous net reclassification improvement (NRI). The “Survival” R package was used to calculate the C-statistic, and the “survIDINRI” R package was used for IDI and NRI. Statistical significance was defined as a two-sided *p*-value < 0.05. All analyses were performed in SPSS (version 26.0, IBM Corporation, Armonk, NY, USA) and RStudio (version 4.4.1, R Foundation for Statistical Computing, Vienna, Austria).

## 3. Results

### 3.1 Baseline Characteristics

**Fig. 2.**
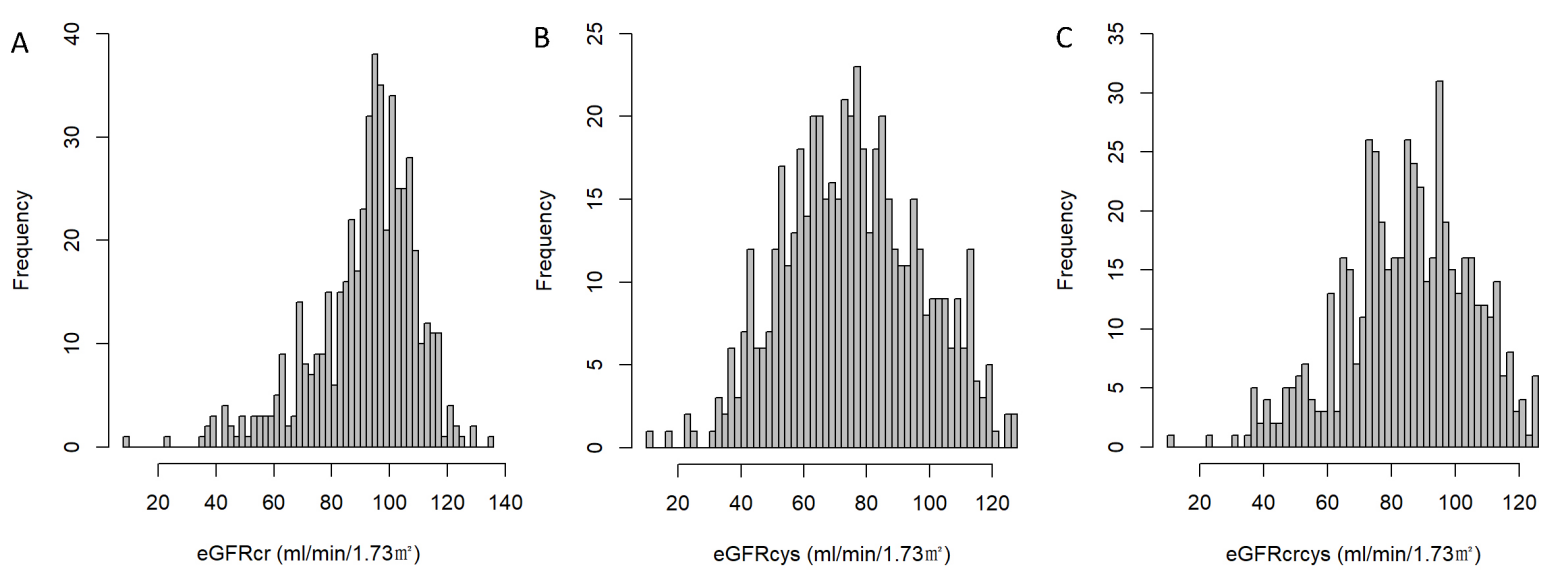
**Distribution of estimated glomerular filtration rate (eGFR) by different equations**. (A) Distribution of eGFR calculated using serum creatinine (eGFRcr). (B) Distribution of eGFR calculated using cystatin C (eGFRcys). (C) Distribution of eGFR calculated by combining creatinine and cystatin C (eGFRcrcys).

After screening, 523 patients were included in the analysis. By the 1-year follow-up, 174 (33.3%) experienced AF recurrence following RFCA. The baseline characteristics and a comparison of the recurrence and non-recurrence groups are summarized in Table 1. The mean age was 60.35 ± 12.16 years, with 191 (36.5%) being female and 217 (41.5%) having persistent AF. The median eGFRcr was 95.09 (83.40, 103.74) mL/min/1.73 m^2^, while mean eGFRcys and eGFRcrcys values were 75.59 ± 21.45 and 84.99 ± 19.71 mL/min/1.73 m^2^, respectively, and their distributions are illustrated in Fig. 2. Notably, eGFRcr exhibited a pronounced right-skewed distribution, whereas eGFRcys approximated a normal distribution.

**Table 1.** **Baseline characteristics of patients**.

		All (n = 523)	Non‐recurrence (n = 349)	Recurrence (n = 174)	*p*
Clinical characteristics					
	Age, years	60.35 ± 12.16	60.06 ± 12.13	60.93 ± 12.25	0.442
	Female gender, n (%)	191 (36.5%)	121 (34.7%)	70 (40.2%)	0.213
	BMI, kg/m^2^	25.41 ± 3.60	25.20 ± 3.46	25.83 ± 3.83	0.057
	Persistent AF, n (%)	217 (41.5%)	134 (38.4%)	83 (47.7%)	0.042
	Duration of AF ≥2 years, n (%)	241 (46.1%)	140 (40.1%)	101 (58.0%)	<0.001
	History of smoking, n (%)	136 (26.0%)	91 (26.1%)	45 (25.9%)	0.958
	History of drinking, n (%)	113 (21.6%)	78 (22.3%)	35 (20.1%)	0.558
Comorbidities					
	Hypertension, n (%)	272 (52.0%)	174 (49.9%)	98 (56.3%)	0.163
	Diabetes mellitus, n (%)	157 (30.0%)	99 (28.4%)	58 (33.3%)	0.243
	Hyperlipidemia, n (%)	124 (23.7%)	80 (22.9%)	44 (25.3%)	0.549
	Coronary heart disease, n (%)	155 (29.6%)	96 (27.5%)	59 (33.9%)	0.131
	Heart failure, n (%)	126 (24.1%)	78 (22.3%)	48 (27.6%)	0.187
	Prior stroke/TIA, n (%)	88 (16.8%)	55 (15.8%)	33 (19.0%)	0.356
Medication					
	Amiodarone, n (%)	188 (35.9%)	120 (34.4%)	68 (39.1%)	0.292
	ACEI/ARB, n (%)	192 (36.7%)	130 (37.2%)	62 (35.6%)	0.718
	Statins, n (%)	193 (36.9%)	127 (36.4%)	66 (37.9%)	0.731
Laboratory test					
	Total cholesterol, mmol/L	3.80 ± 0.97	3.84 ± 0.98	3.74 ± 0.93	0.292
	Triglycerides, mmol/L	1.24 (0.92, 1.71)	1.29 (0.94, 1.75)	1.18 (0.85, 1.63)	0.036
	HDL-C, mmol/L	1.08 (0.90, 1.28)	1.07 (0.91, 1.28)	1.09 (0.89, 1.31)	0.619
	LDL-C, mmol/L	2.26 ± 0.78	2.29 ± 0.80	2.22 ± 0.74	0.325
	Hemoglobin, g/L	138 (126, 149)	139 (128, 149)	136 (121, 146)	0.008
	Fasting blood glucose, mmol/L	5.14 (4.48, 6.07)	5.16 (4.49, 6.08)	5.03 (4.46, 6.03)	0.447
	Hemoglobin A1c, %	5.9 (5.5, 6.5)	5.9 (5.6, 6.5)	5.7 (5.4, 6.5)	0.028
	White blood cell, 10^9^/L	6.00 (5.16, 7.37)	6.03 (5.20, 7.50)	5.90 (5.07, 6.99)	0.227
	ALT, U/L	20 (15, 31)	21 (15, 32)	19 (14, 28)	0.127
	AST, U/L	20 (16, 27)	20 (16, 27)	20 (16, 26)	0.812
	Uric acid, µmol/L	325.77 ± 99.25	325.46 ± 100.07	326.37 ± 97.89	0.921
	eGFRcr, mL/min/1.73 m^2^	95.09 (83.40, 103.74)	95.49 (85.28, 104.01)	93.94 (81.66, 102.37)	0.173
	eGFRcys, mL/min/1.73 m^2^	75.59 ± 21.45	77.77 ± 21.19	71.23 ± 21.35	0.001
	eGFRcrcys, mL/min/1.73 m^2^	84.99 ± 19.71	86.78 ± 19.21	81.40 ± 20.26	0.003
Echocardiographic					
	Left atrial diameter, mm	39.42 ± 6.56	38.87 ± 6.63	40.53 ± 6.30	0.006
	LVED, mm	47.53 ± 5.12	47.40 ± 5.12	47.78 ± 5.13	0.421
	Ejection fraction, %	63 (60, 65)	63 (60, 65)	63 (60, 65)	0.555
	Linear ablation, n (%)	280 (53.5%)	184 (52.7%)	96 (55.2%)	0.597
	SVC isolation, n (%)	43 (8.2%)	30 (8.6%)	13 (7.5%)	0.659
	CHADS_2_ score, n (%)	1 (0, 2)	1 (0, 2)	1 (1, 3)	0.035

Abbreviations: BMI, body mass index; AF, atrial fibrillation; TIA, transient ischemic attack; ACEI, angiotensin converting enzyme inhibitors; ARB, angiotensin receptor blocker; HDL-C, high density liptein cholesterol; LDL-C, low-density liptein cholesterol; ALT, alanine aminotransferase; AST, aspartate aminotransferase; Cr, creatinine; LVED, left ventricular end-diastolic dimension; SVC, superior vena cava.

Compared with those who did not experience recurrence, patients in the recurrence group were more likely to have persistent AF, an AF duration ≥2 years, and higher CHADS_2_ scores (all *p* < 0.05). They also had lower triglyceride, hemoglobin, and hemoglobin A1c levels, as well as reduced eGFRcys and eGFRcrcys, and a larger left atrial diameter (all *p* < 0.05).

### 3.2 Predictive Value of Different eGFR for AF Recurrence

**Fig. 3.**
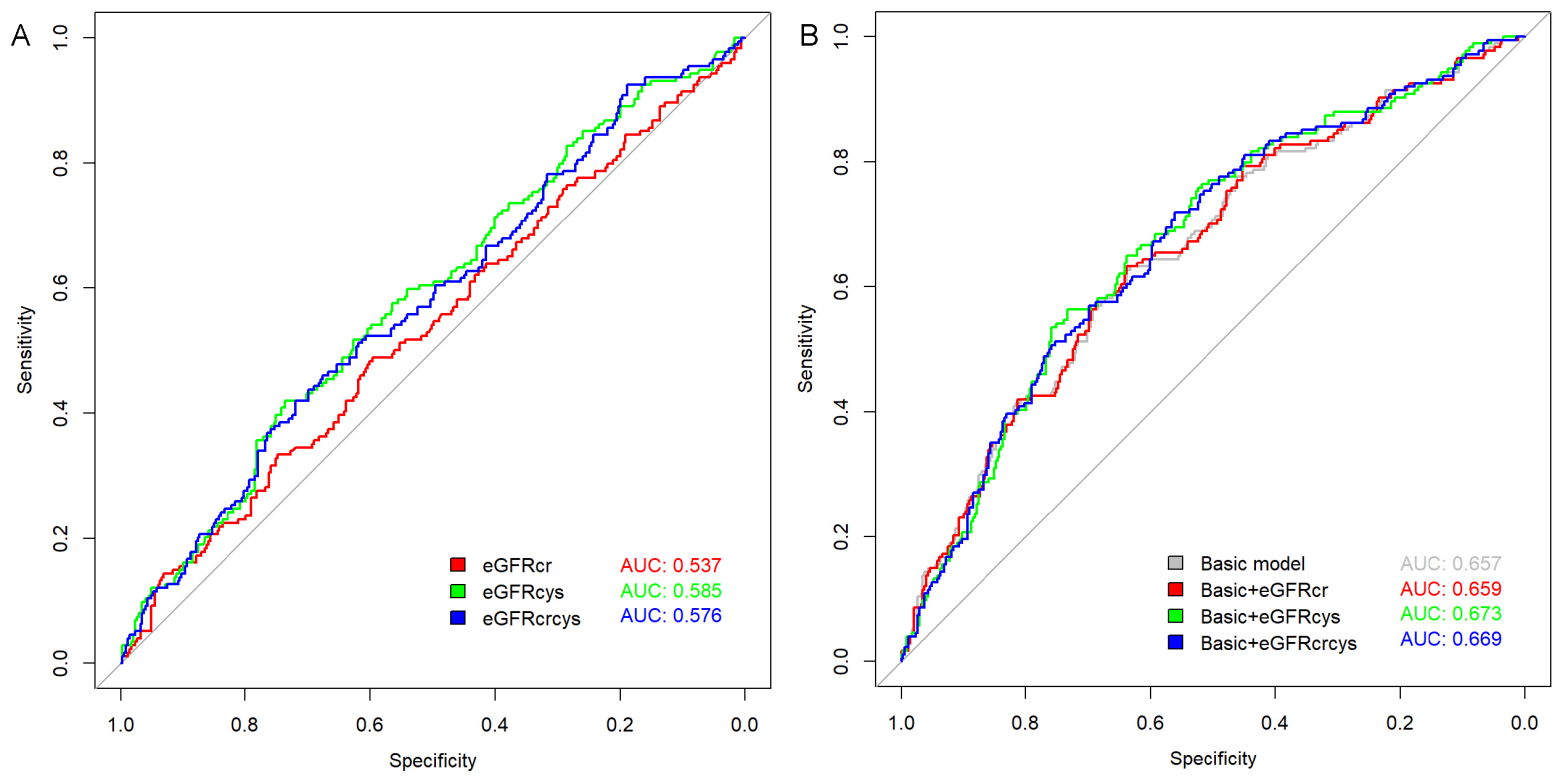
**Receiver operating characteristic (ROC) curves for predicting atrial fibrillation (AF) recurrence**. (A) ROC curves of individual estimated glomerular filtration rate (eGFR) measures: eGFR based on creatinine (eGFRcr), eGFR based on cystatin C (eGFRcys), and combined creatinine-cystatin C eGFR (eGFRcrcys). (B) ROC curves showing the predictive performance when each eGFR measure was added to the basic model.

The predictive value of different eGFR measures for AF recurrence was assessed using ROC analysis (Fig. 3A), revealing an AUC of 0.585 (*p* = 0.001) for eGFRcys, which slightly outperformed both eGFRcr (AUC = 0.537, *p* = 0.179) and eGFRcrcys (AUC = 0.576, *p* = 0.004). The optimal cutoff values were determined as follows: eGFRcr at 92.978 mL/min/1.73 m^2^ (sensitivity 48.9%, specificity 59.6%); eGFRcys at 64.280 mL/min/1.73 m^2^ (sensitivity 42.0%, specificity 73.6%); and eGFRcrcys at 76.093 mL/min/1.73 m^2^ (sensitivity 42.0%, specificity 71.9%).

**Fig. 4.**
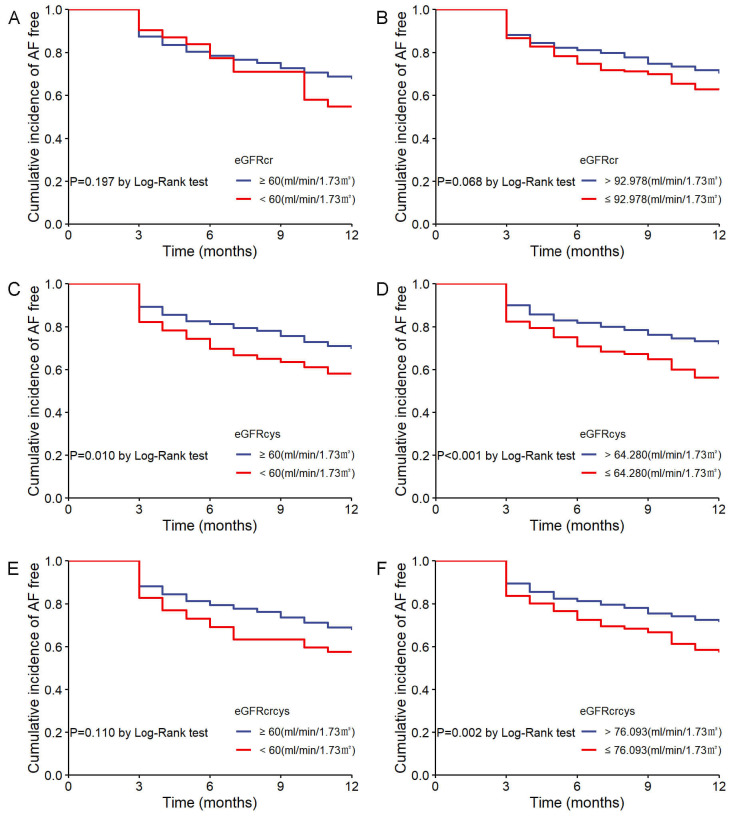
**Cumulative incidence of AF recurrence stratified by eGFR measures**. (A) AF recurrence by eGFRcr with 60 mL/min/1.73 m^2^ cutoff. (B) AF recurrence by eGFRcr with optimal cutoff. (C) AF recurrence by eGFRcys with 60 mL/min/1.73 m^2^ cutoff. (D) AF recurrence by eGFRcys with optimal cutoff. (E) AF recurrence by eGFRcrcys with 60 mL/min/1.73 m^2^ cutoff. (F) AF recurrence by eGFRcrcys with optimal cutoff.

The proportions of patients with eGFRcr, eGFRcys, and eGFRcrcys below 60 mL/min/1.73 m^2^ were 31 (5.9%), 129 (24.7%), and 52 (9.9%), respectively. In the Kaplan-Meier analysis, patients were initially stratified by the 60 mL/min/1.73 m^2^ cutoff, showing no significant difference in AF recurrence for eGFRcr (log-rank *p* = 0.197; Fig. 4A) and eGFRcrcys (log-rank *p* = 0.110; Fig. 4E). However, patients with eGFRcys <60 mL/min/1.73 m^2^ exhibited a significantly higher AF recurrence rate (log-rank *p* = 0.010; Fig. 4C). When stratified by the optimal cutoff values, patients with eGFRcr ≤92.978 mL/min/1.73 m^2^ showed no significant difference in AF recurrence (log-rank *p* = 0.068; Fig. 4B), while those with eGFRcys ≤64.280 mL/min/1.73 m^2^ (log-rank *p* < 0.001; Fig. 4D) and eGFRcrcys ≤76.093 mL/min/1.73 m^2^ (log-rank *p* = 0.002; Fig. 4F) had significantly higher recurrence rates.

### 3.3 Association Between Different eGFR and AF Recurrence

In the univariable Cox regression analyses for the recurrence of AF (**Supplementary Table 1**), persistent AF (HR = 1.364, 95% CI: 1.013–1.837; *p* = 0.041), duration of AF ≥2 years (HR = 1.786, 95% CI: 1.321–2.414; *p* < 0.001), lower hemoglobin levels (HR = 0.988, 95% CI: 0.980–0.996; *p* = 0.005), greater LAD (HR = 1.032, 95% CI: 1.010–1.055; *p* = 0.005), higher BMI (HR = 1.044, 95% CI: 1.002–1.088; *p* = 0.038), and higher CHADS_2_ scores (HR = 1.133, 95% CI: 1.013–1.267; *p* = 0.029) were each significantly associated with AF recurrence. Other parameters examined did not reach statistical significance.

In the multivariable Cox regression analyses (Table 2), when eGFR was treated as a continuous variable, only eGFRcys consistently showed a significant inverse association with AF recurrence across all three models (Model 1: HR = 0.988, 95% CI: 0.981–0.995, *p* = 0.001; Model 2: HR = 0.986, 95% CI: 0.978–0.994, *p* = 0.001; Model 3: HR = 0.990, 95% CI: 0.982–0.998, *p* = 0.019). eGFRcrcys was also significantly associated with AF recurrence in Models 1 and 2 (*p* = 0.003 and *p* = 0.004, respectively) but only showed borderline significance in Model 3 (*p* = 0.067). By contrast, eGFRcr was not significantly associated with AF recurrence.

**Table 2.** **Multivariable Cox regression hazard analyses for the recurrence of AF**.

		Model 1		Model 2		Model 3	
		HR (95% CI)	*p*	HR (95% CI)	*p*	HR (95% CI)	*p*
Continuous							
	eGFRcr	0.994 (0.986–1.002)	0.143	0.995 (0.985–1.004)	0.282	0.999 (0.989–1.009)	0.837
	eGFRcys	0.988 (0.981–0.995)	0.001	0.986 (0.978–0.994)	0.001	0.990 (0.982–0.998)	0.019
	eGFRcrcys	0.989 (0.982–0.996)	0.003	0.987 (0.979–0.996)	0.004	0.992 (0.983–1.001)	0.067
Categorical							
	eGFRcr ≥60 mL/min/1.73 m^2^	Reference		Reference		Reference	
	eGFRcr <60 mL/min/1.73 m^2^	1.416 (0.820–2.446)	0.212	1.266 (0.711–2.257)	0.423	0.983 (0.546–1.771)	0.955
	eGFRcys ≥60 mL/min/1.73 m^2^	Reference		Reference		Reference	
	eGFRcys <60 mL/min/1.73 m^2^	1.524 (1.105–2.101)	0.010	1.541 (1.082–2.194)	0.017	1.287 (0.894–1.853)	0.175
	eGFRcrcys ≥60 mL/min/1.73 m^2^	Reference		Reference		Reference	
	eGFRcrcys <60 mL/min/1.73 m^2^	1.443 (0.923–2.257)	0.108	1.355 (0.843–2.179)	0.210	1.078 (0.660–1.762)	0.764
Categorical by optimal cutoff values							
	eGFRcr >92.978 mL/min/1.73 m^2^	Reference		Reference		Reference	
	eGFRcr ≤92.978 mL/min/1.73 m^2^	1.315 (0.977–1.771)	0.071	1.312 (0.934–1.842)	0.117	1.191 (0.839–1.690)	0.327
	eGFRcys >64.280 mL/min/1.73 m^2^	Reference		Reference		Reference	
	eGFRcys ≤64.280 mL/min/1.73 m^2^	1.743 (1.290–2.356)	<0.001	1.838 (1.313–2.572)	<0.001	1.601 (1.133–2.263)	0.008
	eGFRcrcys >76.093 mL/min/1.73 m^2^	Reference		Reference		Reference	
	eGFRcrcys ≤76.093 mL/min/1.73 m^2^	1.622 (1.200–2.192)	0.002	1.686 (1.200–2.367)	0.003	1.457 (1.026–2.068)	0.036

**Note:** Model 1 did not account for any adjustments. Model 2 was adjusted for age, gender, and BMI. Model 3 was adjusted for age, gender, BMI, AF type, duration of AF ≥2 years, Hemoglobin, LAD, and CHADS_2_ score. HR, hazard ratio; CI, confidence interval.

When categorizing eGFR by 60 mL/min/1.73 m^2^, there was no significant association between any of the three types of eGFR <60 mL/min/1.73 m^2^—eGFRcys, eGFRcr, or eGFRcrcys—and AF recurrence after thorough adjustment. When stratified by the optimal cutoff values, patients with eGFRcys ≤64.280 mL/min/1.73 m^2^ exhibited a significantly higher risk of AF recurrence across all three models (Model 1: HR = 1.743, 95% CI: 1.290–2.356, *p* < 0.001; Model 2: HR = 1.838, 95% CI: 1.313–2.572, *p* < 0.001; Model 3: HR = 1.601, 95% CI: 1.133–2.263, *p* = 0.008). Similarly, patients with eGFRcrcys ≤76.093 mL/min/1.73 m^2^ had a significantly higher recurrence risk across all models (Model 1: HR = 1.622, 95% CI: 1.200–2.192, *p* = 0.002; Model 2: HR = 1.686, 95% CI: 1.200–2.367, *p* = 0.003; Model 3: HR = 1.457, 95% CI: 1.026–2.068, *p* = 0.036). In contrast, patients with eGFRcr ≤92.978 mL/min/1.73 m^2^ did not show a significant increase in AF recurrence risk in any model.

### 3.4 Incremental Predictive Effect of Different eGFR Measures

Adding eGFRcr to the baseline model increases the AUC from 0.657 to 0.659, reflecting a minimal gain in predictive ability. By contrast, incorporating eGFRcys into the baseline model enhances the AUC to 0.673, and eGFRcrcys yields a similar yet slightly lower AUC of 0.669 (Fig. 3B).

As shown in Table 3, the baseline risk model achieves a C-statistic of 0.633 (95% CI: 0.591–0.675). The inclusion of eGFRcr leads to little improvement, with the C-statistic only rising to 0.634 (95% CI: 0.592–0.676) and no significant changes in either IDI (*p* = 0.358) or continuous NRI (*p* = 0.498). By contrast, eGFRcys notably raises the C-statistic to 0.649 (95% CI: 0.608–0.691) and significantly enhances both IDI (*p* = 0.020) and continuous NRI (*p* = 0.030). Similarly, adding eGFRcrcys increases the C-statistic to 0.645 (95% CI: 0.603–0.686) and yields a significant IDI (*p* = 0.020), though its continuous NRI remains non-significant (*p* = 0.109).

**Table 3.** **Added predictive ability and reclassification statistics of eGFR by differing measures**.

	C-statistic (95% CI)	IDI (95% CI)	*p*	Continuous NRI (95% CI)	*p*
Baseline risk model	0.633 (0.591–0.675)	Reference		Reference	
+eGFRcr	0.634 (0.592–0.676)	0.000 (0.000–0.008)	0.358	0.035 (–0.068–0.149)	0.498
+eGFRcys	0.649 (0.608–0.691)	0.008 (0.001–0.024)	0.020	0.076 (0.016–0.173)	0.030
+eGFRcrcys	0.645 (0.603–0.686)	0.005 (0.000–0.019)	0.020	0.075 (–0.027–0.148)	0.109

Baseline risk model: age, gender, BMI, AF type, AF duration ≥2 years, hemoglobin, LAD, and CHADS_2_ score. IDI, integrated discrimination improvement; NRI, continuous net reclassification improvement.

## 4. Discussion

In this study of 523 patients undergoing their first RFCA for AF, we evaluated three eGFR measures (creatinine based, cystatin C based, and a combined equation) to predict AF recurrence within one year. Our findings highlight several key points. First, among these patients with AF, the proportion with eGFRcys below 60 mL/min/1.73 m^2^ (24.7%) was substantially higher than that identified by eGFRcr (5.9%) or eGFRcrcys (9.9%). Second, eGFRcys maintained a consistently stronger inverse relationship with AF recurrence across multiple analytic models, outperforming eGFRcr and showing clearer statistical significance than eGFRcrcys after adjusting for confounders. Moreover, adding eGFRcys or eGFRcrcys to a multivariable model that included traditional risk factors further enhanced the prediction of AF recurrence. Taken together, these observations suggest that the association between eGFR and AF recurrence depends on the specific measurement equation and that eGFRcys appears to provide the strongest predictive value among the three equations.

AF and CKD are closely interconnected, sharing several key risk factors such as hypertension and diabetes [18, 19]. The presence of CKD heightens the likelihood of incident AF, while AF can in turn contribute to the development and progression of CKD [20, 21]. Previous investigations of whether kidney function influences post-ablation AF recurrence have yielded controversial results. Although some observational studies indicate that CKD correlates with higher rates of AF recurrence following ablation, one large cohort study found no independent association between CKD and subsequent AF hospitalization, cardioversion, or repeat ablation [22, 23, 24, 25]. It is worth noting that most of these studies relied exclusively on creatinine-based measurements to define CKD, potentially overlooking early renal impairment. Cystatin C, a sensitive biomarker for kidney function, has been linked in some research to AF recurrence after ablation [26]. A retrospective study involving 183 AF patients undergoing their first ablation found that eGFRcys was a significant predictor of the prevalence of left atrial low-voltage areas [27]. Nevertheless, eGFRcys specifically predicts post-ablation AF recurrence, and how it compares with eGFRcr and eGFRcrcys, remains insufficiently explored. Our findings fill this gap by demonstrating a strong, independent association between preprocedural eGFRcys and post-ablation AF recurrence, whereas eGFRcr does not show a similar relationship.

A recent study found that eight creatinine-based formulas for assessing renal function have varying efficacy in predicting adverse outcomes in patients with AF. However, they did not evaluate the differences between formulas based on creatinine and cystatin C [28]. Multiple studies have explored how different methods of measuring eGFR are utilized in CVD. For instance, prospective cohort analysis of UK Biobank participants identified that eGFRcys was most strongly associated with both CVD and mortality, outperforming traditional creatinine measures and improving existing CVD risk models [11]. Similarly, findings from the Rotterdam Study indicated that lower levels of eGFRcys and eGFRcrcys were significantly linked to higher incident AF risk, whereas eGFRcr decline was not statistically significant [13]. Additional data from the ARISTOTLE trial further underscored the superior prognostic value of cystatin C–inclusive formulas, demonstrating better discrimination for cardiovascular mortality and bleeding in anticoagulated AF patients [29]. Taken together, these findings illustrate a consistent pattern: eGFRcys or combined creatinine-and-cystatin C approaches outperform creatinine-only formulas in predicting cardiovascular events. Aligned with these outcomes, our results reinforce the notion that cystatin C-based eGFR is more effective for stratifying the recurrence risk of RFCA in AF patients than creatinine-based estimations alone.

Potential explanations for the distinct relationship between different eGFR measures and AF recurrence following RFCA remain unclear. However, differences between cystatin C and creatinine may explain this phenomenon. Creatinine is widely influenced by age, muscle mass, diet, and physical activity, which may lead to an overestimation of the actual GFR when using eGFRcr [30, 31]. By contrast, cystatin C is produced at a relatively constant rate and is less influenced by these factors, making it more sensitive to early or mild renal impairment [32]. This contrast is also evident in our findings, where eGFRcys identified a larger proportion of CKD G3–5 patients than did eGFRcr or eGFRcrcys.

Several potential limitations should be recognized. First, due to the retrospective nature of this study and the fact that cystatin C was measured in only a small subset of patients, there is a potential for selection bias. Second, although we adjusted for numerous variables, residual confounding may still exist. For example, while we included LAD as a covariate, left atrial volume may be a more accurate measure of left atrial size. Third, eGFR was measured only once prior to ablation, and fluctuations in kidney function over time were not captured. Fourth, although our follow-up protocol included electrocardiogram and 24-hour Holter monitoring at predefined intervals, asymptomatic recurrences could have been underdetected. Lastly, our conclusions regarding their applicability to populations using different ablation methods and strategies still require further investigation. Thus, large-scale prospective studies are necessary to further validate our findings.

## 5. Conclusions

This study suggests significant differences in the predictive value of various eGFR measures for AF recurrence after RFCA. eGFRcys demonstrated a stronger independent association with AF recurrence compared to eGFRcr. These findings emphasize the importance of utilizing eGFRcys for effective risk stratification in AF patients. Incorporating this measure into clinical practice may enhance individualized management strategies and improve outcomes for patients undergoing RFCA.

## Data Availability

All data generated or analyzed during this study are included in the article, and the data supporting the findings of this study are available on request.

## Abbreviations

AF, atrial fibrillation; AUC, area under curve; CKD, chronic kidney disease; CVD, cardiovascular disease; eGFR, estimated glomerular filtration rate; IDI, integrated discrimination improvement; LAD, left atrial diameter; NRI, continuous net reclassification improvement; RFCA, radiofrequency catheter ablation; ROC, receiver operating characteristic.

## Supplementary Material

Supplementary material associated with this article can be found, in the online version, at https://doi.org/10.31083/RCM42848.
