# Supplementary material for: Glomerular Filtration Rate by Differing Measures in Predicting Atrial Fibrillation Recurrence After Ablation
Source: Rev Cardiovasc Med. 2025 Dec 18;26(12):42848. doi: 10.31083/RCM42848 (PMC12781001; doi:10.31083/RCM42848)
Supplement: Supplementary file 1 [file 2153-8174-26-12-42848-s1.docx]

Supplementary Table 1. Univariable Cox regression hazard analyses for AF recurrence

|  | HR | 95%CI | *p* |
| --- | --- | --- | --- |
| Age, years | 1.004 | 0.991–1.016 | 0.569 |
| Female gender | 1.197 | 0.884–1.621 | 0.244 |
| BMI, kg/m^2^ | 1.044 | 1.002–1.088 | 0.038 |
| Persistent AF | 1.364 | 1.013–1.837 | 0.041 |
| Duration of AF≥2 years | 1.786 | 1.321–2.414 | <0.001 |
| History of smoking | 0.997 | 0.710–1.399 | 0.984 |
| History of drinking | 0.911 | 0.629–1.320 | 0.622 |
| Hypertension | 1.211 | 0.897–1.634 | 0.211 |
| Diabetes mellitus | 1.194 | 0.871–1.636 | 0.271 |
| Hyperlipidemia | 1.089 | 0.774–1.533 | 0.625 |
| Coronary heart disease | 1.249 | 0.913–1.710 | 0.164 |
| Heart failure | 1.233 | 0.884–1.720 | 0.217 |
| Prior stroke/TIA | 1.208 | 0.827–1.765 | 0.328 |
| Amiodarone | 1.191 | 0.879–1.616 | 0.260 |
| ACEI/ARB | 0.955 | 0.700–1.303 | 0.772 |
| Statins | 1.048 | 0.772–1.423 | 0.764 |
| TC, mmol/L | 0.914 | 0.784–1.067 | 0.255 |
| TG, mmol/L | 0.831 | 0.675–1.023 | 0.080 |
| HDL-C, mmol/L | 1.159 | 0.709–1.893 | 0.556 |
| LDL-C, mmol/L | 0.905 | 0.749–1.095 | 0.304 |
| Hemoglobin, g/L | 0.988 | 0.980–0.996 | 0.005 |
| Fasting blood glucose, mmol/l | 0.988 | 0.916–1.066 | 0.763 |
| Hemoglobin A1c, % | 0.934 | 0.792–1.102 | 0.417 |
| WBC, 10^9^/L | 0.941 | 0.866–1.023 | 0.152 |
| ALT, U/L | 0.991 | 0.981–1.000 | 0.053 |
| AST, U/L | 0.995 | 0.982–1.008 | 0.434 |
| UA, umol/L | 1.000 | 0.999–1.002 | 0.819 |
| LAD, mm | 1.032 | 1.010–1.055 | 0.005 |
| LVED, mm | 1.010 | 0.982–1.039 | 0.478 |
| LVEF, % | 1.001 | 0.983–1.019 | 0.933 |
| Linear ablation | 1.098 | 0.814–1.480 | 0.541 |
| SVC isolation | 0.900 | 0.512–1.585 | 0.716 |
| CHADS_2_ score | 1.133 | 1.013–1.267 | 0.029 |

Abbreviations: AF, atrial fibrillation; BMI, body mass index; ACEI, angiotensin converting enzyme inhibitors; ARB, angiotensin receptor blocker; TC, total cholesterol; TG, triglycerides; HDL-C, high density liptein cholesterol; LDL-C, low-density liptein cholesterol; WBC, white blood cell; ALT, alanine aminotransferase; AST, aspartate aminotransferase; UA, uric acid; LAD, left atrial diameter; LVED, left ventricular end diastolic diameter; LVEF, left ventricular ejection fraction.
